# Automatic determination of mercury in samples of environmental interest

**DOI:** 10.1155/S1463924696000235

**Published:** 1996

**Authors:** Maria Cristina Canela, Wilson F. Jardim, Jarbas J. R. Rohwedder

**Affiliations:** Instituto de Química Universidade Estadual de Campinas CEP 13083-970 Campinas São Paulo CP 6154 Brazil


Journal of Automatic Chemistry, Vol. 18, No. 6 (November–December 1996) pp. 193–198

### Automatic determination of mercury in samples of environmental interest

**Maria Cristina Canela, Wilson F. Jardim and Jarbas J. R. Rohwedder**

*Instituto de Química, Universidade Estadual de Campinas, CEP 13083-970, CP 6154, Campinas, São Paulo, Brazil*

*An automatic flow injection (FI) system for the determination of mercury was developed using a commercial cold vapour atomic absorption spectrophotometer (CVAAS). Control and data acquisition in the FI system was done with an IBM-PC 286 XT compatible microcomputer and a home-made interface, using software written in QuickBasic 4·5. Mercury content was determined by: sampling using a combination of four electromechanical three-way poly(tetrafluoroethylene) valves; separation of the dissolved reduced mercury in a gas/liquid separation cell using nitrogen as carrier, followed by amalgamation of the stripped metal on a gold wire column; after stripping the metal, cleaning the separation cell using vacuum, which was controlled by a three-way electromechanical valve; heating the gold wire column automatically to release the amalgamated mercury using an external nichrome wire coil; storing the output signals automatically to calculate the final mercury concentration, using commercially available software. The optimized system presents a detection limit of 5·3 ng $l^{-1}$ of mercury (30 pg absolute) using 5·7 ml (three injections of 1900 μl of the sample) with an analytical frequency of six samples per hour and reproducibility of 5%. The procedure was used to determine mercury in fish, hair and natural water samples.*

### Introduction

The effects of environmental contamination caused by mercury have been known for decades [1]. Mercury can be introduced into the environment by both natural and anthropogenic sources, including volcanic emissions, mining, industrial and agricultural activities [2, 3]. To assess and evaluate the real impact of mercury in the environment, it is necessary to quantify the metal stock in a large variety of discrete reservoirs, as well as the flux among these systems. In natural waters, for example, levels below the detection limit of most analytical techniques are often encountered. Typical values for total dissolved mercury concentration are at the picomolar level (0·2–100 ng $l^{-1}$) and, in some cases, are even lower [4, 5].

Methods used for mercury determination include colorimetry, atomic absorption spectrophotometry, cold vapour atomic absorption spectrophotometry (CVAAS), cold vapour atomic fluorescence spectrophotometry (CVAFS) and neutron activation analysis [6]. Among these methods, CVAAS and CVAFS are currently the most widely used mainly due to their very low limits of detection. CVAAS was first described by Polucktov and Vitkun in 1963, and further developed by Hatch and Ott [7, 8]. Since then, numerous modifications and adaptations have been reported in the literature [7, 9–21]. These modifications include adaptation of the cold vapour to a flow injection (FI) system [11, 16–18], the use of chromatography columns for speciation [19], utilization of gold filled columns for one or two stage mercury amalgamation [10, 13, 14, 20] and, finally, by automation of the analyser itself [9, 21].

Pre-concentration procedures have a high level of reliability and applicability due to the lower detection limit [10, 13, 22]. In this procedure a gold and mercury ($Hg^0$) amalgam is formed after stripping the reduced form of the metal from the matrix. After thermal desorption, mercury vapour is carried into the detection cell using a stream of inert gas. However, this pre-concentration procedure increases the number of analytical steps involved which makes the manual procedure difficult, so, automated systems to replace manual procedures are now becoming more popular.

This paper describes the development and optimization of an automatic system used in the determination of mercury in samples of environmental interest using CVAAS. Mercury was pre-concentrated in a gold filled column, which was adapted to the FI system by injecting the same discrete volume of sample up to three times.

### Experimental

### *Reagents*

All reagents were of analytical grade and deionized water (Milli-Q) was used throughout. Glassware was cleaned by soaking overnight in 10% v/v nitric acid, followed by meticulous rising in deionized water.

The reductant solution was a mixture (6:4 v/v) of a nitric acid (5% v/v) solution and a tin (II) chloride (10% w/v) solution. This solution was purged with nitrogen for 15 min before use to eliminate any contamination of $Hg^0$.

Working standard solutions of mercuric ions (from 0·01 up to 100 ppb) were prepared by appropriate dilution of a stock solution containing 1000 mg $l^{-1}$ of the metal (Merck). These solutions (50 ml) were prepared daily, and preserved with 1 ml of concentrated nitric acid and 25 μl of 0·01 g $ml^{-1}$ potassium dichromate.

### *Automatic FI system*

The automatic FI system for the determination of mercury is shown in figure 1. PTFE tubing of 0·8 mm i.d. was used for the manifold.

0142-0453/96 $12·00 © 1996 Taylor & Francis Ltd

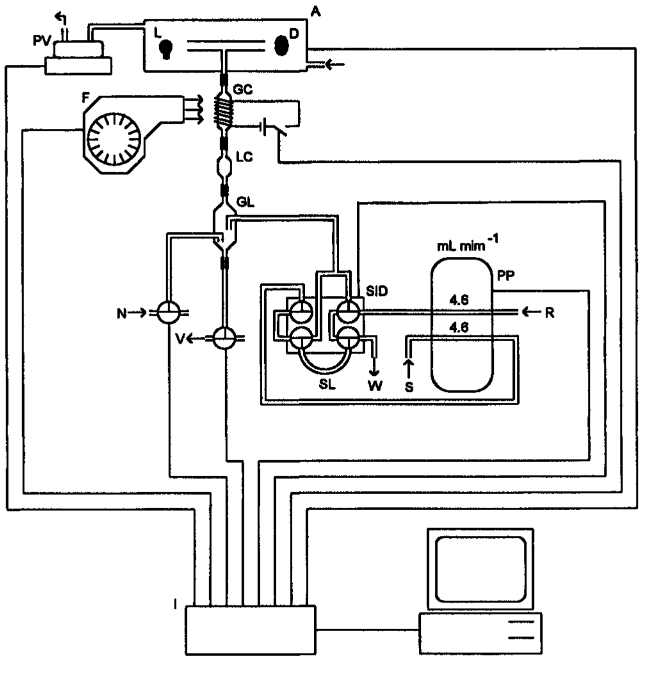

*Figure 1. Automatic system for mercury determination. A = CVAAS; D = detector; F = fan; GC = gold column; GL = gas/liquid separation; I = interface; L = lamp; LC = lime column; N = nitrogen; PP = peristaltic pump; PV = vacuum pump; R = reagents; S = sample loop; SID = sample introduction device; V = vacuum; and W = waste.*

A Bück Scientific-400-A cold vapour absorption mercury analyser with an open Pyrex T-shaped cell (150 mm long with an internal diameter of 7 mm) was used to detect the mercury vapour [11, 23]. The automated version was based on a prototype developed by Pasquini *et al.* [11].

An Ismatec MS-REGLO-7331-00 four-channel peristaltic pump fitted with Viton pumping tubes was used for fluid flow control.

An IBM-PC 286 XT compatible microcomputer with 640 kbyte RAM, 20 Mbyte Winchester, 360 kbyte floppy, and a CGA-monochrome display was used for control and data acquisition in the automatic FI system.

*Interface*

The control device of the FI system was done by using an 8 bit parallel asynchronous interface [24, 25]. The interface communicates with the microcomputer through a user port based on the CI 8255 [25], which is plugged into an extension slot inside the microcomputer.

The dynamic analogue-to-digital converter described previously [25] was replaced by the 8 bit analogue-to-digital converter (ADC 0808) for data acquisition of the CVAAS output.

Three of the output lines of an 8 bit latch (CI 74LS373) [25] were used to source the base current to TIP 121 transistors which controlled the electromechanical valves. Another four lines of this latch were used to control four solid state relays, used to switch the peristaltic pump, the cooling fan, the heating coil of the gold column, and the CVAAS internal vacuum pump on and off.

*Gas/liquid separation cell*

The gas/liquid separation cell is shown in figure 1. The cell used in this work was modified from the one previously described [11]. The cell was constructed of Pyrex and two different sizes were tested. When using a 550 µl sample loop, a 5·0 cm long and 1·0 cm internal diameter cell was used. When a 1900 µl sample loop was used, the separation cell measured 2·0 cm inner diameter and 6·0 cm in length.

*Gold and lime soda column*

The gold amalgamation column was constructed using a 4·0 cm long and 0·35 cm inner diameter quartz tube. The column was filled with randomly twisted gold wire (0·21 mm diameter-Degussa, 200 mg). The column was heated by a 1 m external coil of nichrome wire ($R = 38{\cdot}0\ \Omega\ m^{-1}$).

A pre-column, to protect the gold column from acids and water vapours, was constructed using a 2·5 cm long and 0·35 cm inner diameter glass tube, filled with ~70 mg of soda lime (Darex), placed between small plugs of glass wool.

*Sample introduction device*

Figure 2 shows the sample introduction device used in the automatic FI system. This device is a modification of a similar one previously [26] described, where a fourth electromechanical three-way valve (NResearch-161T301, 12 V, 80 mA) was included. This modification allows the sample to be aspirated by the peristaltic pump, rather than by gravity [26].

A control signal, generated by the microcomputer, changed the device from sampling to injection mode by simultaneously turning all valves on or off, respectively.

*Software to control the automatic FI system*

The software to drive the users' port and interface has been described previously [27, 28]. In addition, to control the automatic FI system, a sub-program was written in QuickBasic 4·5 (see the flow chart in figure 3). Cyclic operation of the system starts by turning the peristaltic pump on. During this stage, the sample introduction device is in the sampling position (all valves off). The reductant solution is continuously pumped and removed from the gas/liquid separation cell by vacuum. When the selected sample loop has been filled with sample solution, the sampling device valves are turned on, the vacuum valve is turned off, and the nitrogen control valve is turned on. Now the reductant solution carries the sample to the separation cell where nitrogen is being purged continuously into the separation cell.

After the whole sample loop has been introduced into the gas/liquid separation cell, the pump and sample introduction device are turned off. After complete removal of elemental mercury from the solution, the nitrogen stream

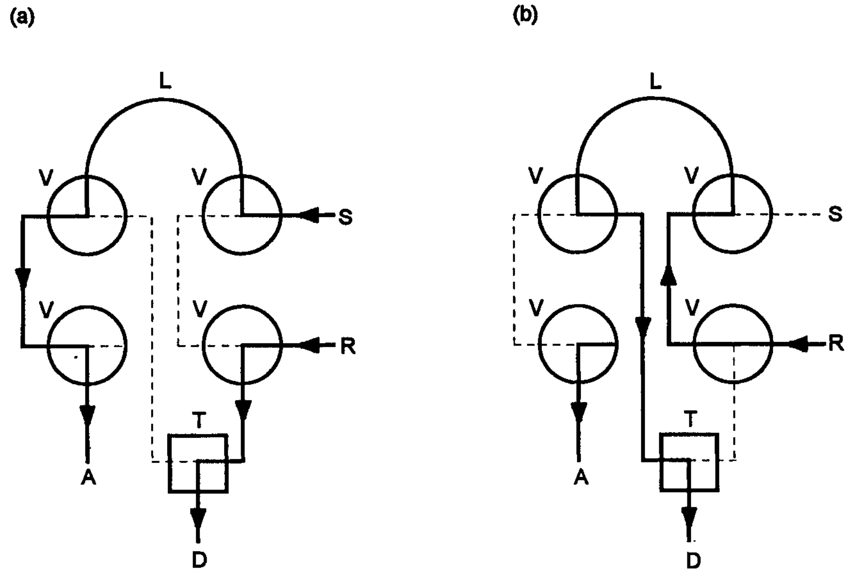

*Figure 2. Sample introduction device diagram. L = sample loop; S = sample inlet; A = sample aspiration; R = reagents; D = to detector; V = electromechanical three way valve; T = 'T'-shaped connector.*

is closed. The mercury vapour generated during this process is pre-concentrated onto the gold-filled column. The gas/liquid separation cell is subsequently emptied, and the vacuum turned off. The column is then heated at 320 °C for 30 s. After this time interval, heating is turned off and nitrogen is turned on. Simultaneously, the data acquisition routine, converting analogue to digital signals, starts. After 30 s of data collection, the CVAAS internal vacuum pump, as well as the cooling fan of the gold filled column, starts to operate, and the detection cell is cleaned within 75 s. As shown in figure 3, the program also allows the use of the pre-concentration step alone. The signal obtained is displayed on the microcomputer screen and simultaneously saved onto the hard disk.

### *Determination of mercury in fish, hair and natural waters*

Determinations of total mercury in fish and hair samples were carried out using certified samples supplied by the Chemistry Department of University of Brasília, Brazil. Suitable amounts of samples were digested using 8 ml of concentrated nitric acid and 2 ml of distilled water. The solution was heated for approximately 30 minutes at 90 °C and later diluted to 10 ml using deionized water. Mercury was determined in these samples without a pre-concentration step, in which case the flow chart presented in figure 3 was partially followed.

Water samples were collected around Campinas (from the Atibaia, Anhumas, and Jundiaí rivers), as well as in the Amazon region (from the Igarapé do Inácio and Igarapé do Pretinho rivers). Samples were preserved using 4 ml of concentrated $HNO_3$ for every 1 l of sample. Digestions were carried out in a conventional microwave oven using 5 ml of sample with 5 ml of concentrated nitric acid. The 10 ml mixture was put in PTFE bottles and heated for 5 min at moderate power (350 W). The digested solutions were diluted to 25 ml and analysed as soon as the digestion procedure finished.

### Results and discussion

In order to achieve a lower mercury detection limit than that previously reported by Pasquini et al. [11], the original system was modified by introducing the gold amalgamation column before CVAAS quantification. However, when manually operated, the procedure involving this pre-concentration step showed poor reproducibility: this was mainly due to the large number of operations necessary for each determination. Therefore, it became necessary to develop an automatic system to control the various steps, from sampling up to transmittance readings. The automated system was then developed and optimized for the following parameters: sampling loops of 550 µl and 1900 µl; the flow of gas carrier; sampling time; injection time, and purge time. The best values obtained are presented in table 1.

Perhaps the most critical experimental parameter in the whole procedure is setting the appropriate gas flow rate. The carrier gas is responsible not only for the $Hg^0$ transference from the liquid to the gas phase in the separation cell, but it also carries the elemental mercury into the detection cell with such a flow rate that the highest metal density is achieved during the atomic absorbance readings.

*Table 1. Best values for automatic system.*

| | | |
|---|---|---|
| Sampling loop/µl | 550 | 1900 |
| Gas flow/ml $min^{-1}$ | 70 | 93 |
| Sampling time/s | 20 | 40 |
| Injection time/s | 15 | 50 |
| Purge time/s | 30 | 45 |

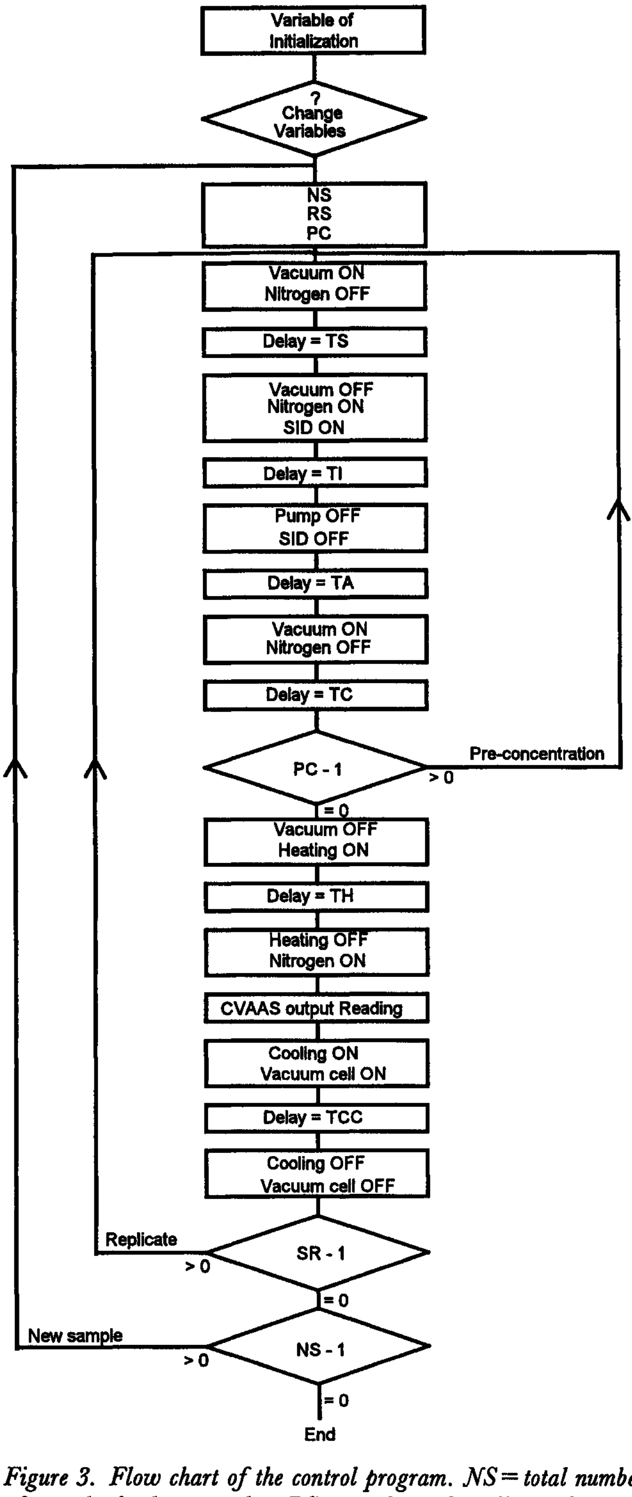

*Figure 3. Flow chart of the control program. NS = total number of standard plus sample; RS = number of replicate for each sample; PC = number of pre-concentration steps for each sample; TS = sampling time; TI = sample introduction time; TA = amalgamation timer; TC = cleaning of the gas/liquid separation cell; TH = heating of the gold column timer; TCC = cooling of the gold column and cleaning of the chamber of the detector cell; SID = sample introduction device.*

Amalgamation column heating (30 s) and cooling (75 s) time lengths were not altered by changing the injected sample volume. The temperature achieved inside the column under the described conditions was 320 °C: enough to strip out all amalgamated mercury. The cooling time was necessary to restore the column to room temperature before starting a new cycle of sample processing.

*Table 2. Detection limit and injection frequency.*

| Loop/μl | No. of injections | DL*/ng $l^{-1}$ | Samples/h |
|---|---|---|---|
| 550 | 1 | 37·5 | 19 |
| | 2 | 36·0 | 12 |
| | 3 | 26·6 | 10 |
| | | DL/ng $l^{-1}$ | Samples/h |
| 1900 | 1 | 18·3 | 12 |
| | 2 | 7·9 | 8 |
| | 3 | 5·3 | 6 |

* Detection limit

After these conditions were established, further studies centred on the determination of the detection limit of the system; the values obtained are shown in table 2. As expected, the results show that an increase in sampling volume reflects in a decrease in the detection limit. The same trend was observed when several stages of amalgamation were performed using the same loop, instead of using fewer injections but with a loop holding more sample.

Figure 4 shows the signal obtained for a calibration curve using a 1900 μl sampling loop and two pre-concentration cycles in the range of 10 up to 75 ng $l^{-1}$ of mercury.

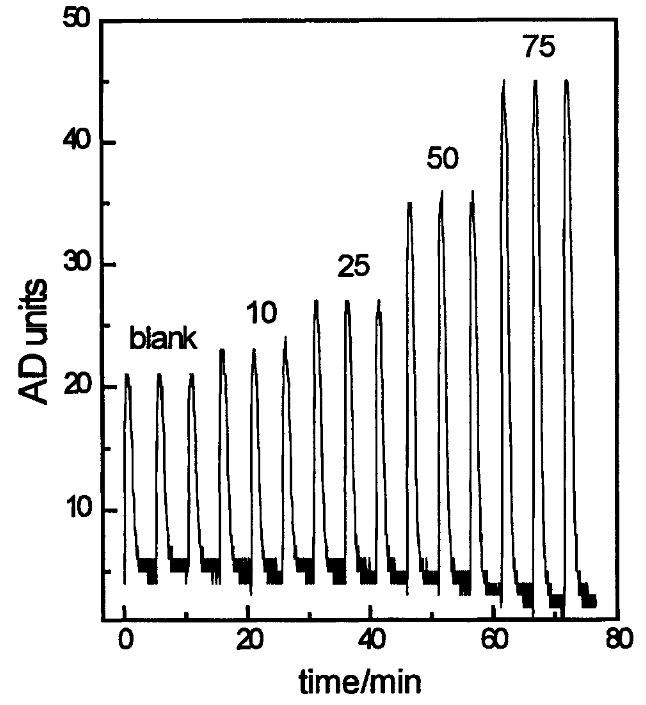

*Figure 4. Typical chart recorded signals. A blank and four standard solutions. The numbers represent mercury concentrations in ng $l^{-1}$. All signals shown are for triplicate injection of the same solution.*

*Table 3. Mercury concentration found in natural water samples.*

| Sample | Hg inorganic/ng $l^{-1}$ | Hg total/ng $l^{-1}$ |
|---|---|---|
| Atibaia river | 15·4 | 148·0 |
| Jundiaí river | <5·0 | 217·0 |
| Córrego Anhumas | <5·0 | 295·0 |
| Igarapé do Inácio | <5·0 | 32·2 |
| Igarapé do Paulista | <5·0 | 40·4 |

* Using three pre-concentration steps.

*Table 4. Results obtained for total mercury in hair and fish and standard deviation*

| Laboratory | Hair—Hg/μg $g^{-1}$ | Fish—Hg/μg $g^{-1}$ |
|---|---|---|
| I | 7·60 ± 0·6 | 6·20 ± 0·8 |
| II | 6·10 ± 0·3 | 4·70 ± 0·3 |
| III | 7·61 ± 0·1 | 6·10 ± 0·3 |
| IV | 7·48 ± 0·2 | 6·72 ± 0·2 |
| This work | 7·01 ± 1·5 | 6·20 ± 0·6 |

Output signals show good reproducibility, with a relative standard deviation of 5% ($N = 3$). The curve was described by the equation $y = 0{\cdot}38 \times -2{\cdot}72$, where $y$ is the peak height (in arbitrary units) and $x$ is the mercury concentration (ng $l^{-1}$) with a correlation coefficient of 0·996. The high signal obtained for the blank was attributed to the use of analytical grade reagents, rather than supra pure grade.

With the introduction of the gold filled column in the system, it was possible to determine mercury in natural waters. The results obtained for water samples are presented in table 3. The concentration of reactive mercury, defined as the amount of mercury quantified after reduction with Sn (II), in natural waters was below the detection limit of the system. On the other hand, total mercury concentration obtained for digested samples showed metal concentrations well above the detection limit of the method.

Fish and hair samples were determined as part of a mercury intercalibration programme in the Brazil, and the results obtained are shown in table 4. These values agree with those obtained by five other laboratories.

### Conclusion

The results showed that the introduction of an amalgamation column and the automation of the system improved the detection limit for mercury, making it feasible to determine levels of 10 ng $l^{-1}$, which is 20 times lower than the detection limit obtained using the previously described method [11].

According to Corns *et al.* [18], CVAAS is not the most sensitive technique for mercury determination. However, the present work reports similar values of detection limit as the one obtained using CVAFS. Stockwell [21], using a completely automatic system coupled to CVAFS, but running 40 samples per hour, obtained the same detection limit as the one reported in this paper. Winfield *et al.* [15] also obtained a detection limit of 10 ng $l^{-1}$ using CVAFS and two gold columns.

More recently, methods based upon atomic fluorescence, with detection limits ranging from 0·001 to 1 ng $l^{-1}$ of mercury have been developed [9, 29, 30]. However, there are still some advantages when using the system developed in this work, especially when dealing with biological fluids, where large amounts of sample are not always available. For instance, some authors have also determined mercury at a level of 10 ng $l^{-1}$, but to achieve this, a volume of approximately 50 ml of the sample was necessary [10, 19, 21]. The low cost of the detector is another advantage of the proposed system when compared to CVAFS and graphite furnace AAS.

Another important aspect of the proposed method is the versatility of the analytical working range. Depending on the concentration of mercury in a given sample, the system can be easily modified by changing the sampling loop, removal of the amalgamation column, or even by choosing the appropriate subroutine to extend the working range.

The major disadvantage of the proposed system is the low analytical rate when compared to other methods [21], as well as the high memory effect observed when working with samples showing a wide range of mercury concentration. In this case, an additional cleaning for decontamination of the system is necessary, thus decreasing the number of samples performed per hour. Nonetheless, this behaviour is also believed to occur with any other method suitable to trace analysis.

### Acknowledgement

The authors are grateful Dr C. H. Collins for revision of the manuscript and to FAPESP for financial support.

### References

1. Fitzgerald, W. F. and Clarkson, T. W., *Environmental Health Perspectives*, **96** (1991), 159.

2. Clarkson, T. W., *Environmental Health Perspectives*, **100** (1992), 31.

3. Osa, R. H. (Ed), *Mercury Atmospheric Processes—A Synthesis Report: Workshop Proceedings* (MES, Tampa, Florida, 1994).

4. Vandal, G. M. *et al.* (Eds), *The Production and Evasion of Elemental Mercury in Lakes: a Study of Palette Lake, Northern Wisconsin, USA* (CEP Consultants Ltd, Toronto).

5. Bloom, N. S. and Crecelius, E. A., *Marine Chemistry*, **14** (1983), 49.

6. Mitra, S., *Mercury in the Ecosystem—Its Dispersion and Pollution Today* (Transtech Publication Ltd, Suiça, 1986).

7. Corns, W. T., Ebdon, L. C., Hill, S. J. and Stockwell, P. B., *Analyst*, **117** (1992), 717.

8. Daniels, R. S. and Wigfield, D. C., *Analytica Chimica Acta*, **248** (1991), 575.

9. Cossa, D., Sanjuan, J., Cloud, J., Stockwell, P. B. and Corns, W. T., *Journal of Analytical Atomic Spectrometry*, **10** (1995), 287.

10. Liang, L. and Bloom, N. S., *Journal of Analytical Atomic Spectrometry*, **8** (1993), 591.

11. Pasquini, C., Jardim, W. F. and Faria, L. C., *Journal of Automatic Chemistry*, **10** (1988), 188.

12. Temmerman, E., Van de casteele, C., Vermier, G., Leyman, R. and Dams, R., *Analytica Chimica Acta*, **236** (1990), 371.

13. Temmerman, E., Dumarey, R. and Dams, R., *Analytical Letters*, **18** (1990), 203.

14. Zachariadis, G. A. and Stratis, J. A., *Journal of Analytical Atomic Spectrometry*, **6** (1991), 239.

15. Winfield, S. A., Boyd, N. D., Vimy, M. J. and Lorscheider, F. L., *Clinical Chemistry*, **40** (1994), 206.

16. Andrade, J. C., Pasquini, C., Baccan, N. and Van Loon, J. C., *Espectrochimica Acta*, **38B** (1983), 1329.

17. Oda, C. E. and Ingle, Jr., J. D., *Analytical Chemistry*, **53** (1981), 2030.

18. Corns, W. T., Edbon, L. C., Hill, S. J. and Stockwell, P. B., *Journal of Automatic Chemistry*, **13** (1991), 267.

19. Bloom, C., *Canadian Journal Fisheries Aquatic Science*, **46** (1989), 1131.

20. Fitzgerald, W. F. and Gill, G. A., *Analytical Chemistry*, **51** (1979), 1714.

21. Stockwell, P. B.., *Journal of Automatic Chemistry*, **16** (1994), 155.

22. Friese, K. H., Roschig, M., Wuenscher, G. and Matschiner, H., *Fresenius Journal Analytical Chemistry*, **337** (1990), 860.

23. Goto, M., Shibakawa, T., Arita, T. and Ishu, D., *Analytica Chimica Acta*, **140** (1982), 179.

24. Souza, P. S. and Pasquini C., *Laboratory Micrompute*r, **9** (1990), 77.

25. Cunha, I. B. S. and Pasquini, C., *Analyst*, **117** (1992), 905.

26. Pasquini, C. and Faria, L. C., *Journal of Automatic Chemistry*, **13** (1991), 143.

27. Malcome-Lawes, D. J., *Laboratory Microcomputer*, **6** (1987), 16.

28. Malcome-Lawes, D. J., *Laboratory Microcomputer*, **6** (1987) 122.

29. Corns, W. T., Stockwell, P. B. and Jameel, M., *Analyst*, **119** (1994), 2481.

30. Janjic, J. and Kiurski, J., *Water Research*, **28** (1994), 233.

